# Using Traditional Chinese Medicine to Treat Hepatocellular Carcinoma by Targeting Tumor Immunity

**DOI:** 10.1155/2020/9843486

**Published:** 2020-06-11

**Authors:** Wentao Jia, Lina Wang

**Affiliations:** School of Traditional Chinese Medicine, Naval Medical University, Xiangyin Road No. 800, Shanghai 200433, China

## Abstract

As the leading cause of cancer-related death, hepatocellular carcinoma (HCC) threatens human health and limited treatments are available to cure the disease efficiently and effectively. The particularly immunotolerant environment of the liver lowers the efficacy of current therapies in patients with advanced HCC. Traditional Chinese medicine (TCM) is gathering increasing interest due to the immunoregulatory properties of certain compounds. In advanced HCC, TCM can restore immunosurveillance to promote antitumor effects in several ways, including the upregulation of immunostimulatory factors and the downregulation of immunosuppressive factors. The characteristic multitarget regulation of TCM compounds may provide new insights regarding effective HCC immunotherapies. Here, we review the immunoregulatory potency of TCMs for treating HCC and explain how individual TCM drugs and complex formulas remodel the immune environment in various cell- and cytokine-dependent manners.

## 1. Introduction

As the second leading cause of cancer-related death worldwide and the third deadliest cancer in China, liver cancer poses a serious threat to human health [1]. Recent epidemiological data indicate that ∼4,292,000 people in China are newly diagnosed with liver cancer each year and that ∼2,814,000 patients die from the disease annually [1]. The liver is a susceptible organ for both primary and metastatic cancer. The main pathological forms of primary liver cancer include hepatocellular carcinoma (HCC), intrahepatic cholangiocarcinoma, and a mixed type, with HCC being the most prevalent [2–4]. HCC tumors possess a high degree of heterogeneity, and, as such, this form of cancer is associated with poor prognosis and inefficient clinical treatment, with a 5-year survival rate of only 3%–5% [5]. Currently, radical methods for treating early stage HCC (<3 cm) include liver resection, liver transplantation, and radiofrequency ablation. Radical treatment methods are still limited for advanced HCC [5]. Although molecular targeted therapies that have been approved by the United States Food and Drug Administration (FDA), such as sorafenib and regorafenib, can have beneficial effects for patients with advanced HCC, the increase in median survival time is still less than 3 months [5, 6]. Moreover, severe drug resistance against molecular targeted therapy is maintained over long-term applications [7]. Recently, novel treatments, including immunotherapy and oncolytic therapy, have demonstrated marvelous curative efficiency and have brought new hope for treating several advanced cancers. Specifically, recent immunomodulatory strategies can be classified as adoptive immunotherapies (based on natural killer (NK) and cytokine-induced killer cells), indirect immunological strategies (based on cytokines, immune checkpoint inhibitors, and vaccines), and indirect nonimmunological strategies (based on metronomic chemotherapy and oncolytic viruses) [8]. In 2017, a programmed death-1 (PD-1) inhibitor—nivolumab—was approved by the FDA to treat HCC patients who had undergone sorafenib treatment; this milestone, based on the pivotal conclusion of the CheckMate 040 clinic trial, marked HCC immunotherapy as an emerging hot field [9, 10]. Subsequently, several therapies, including immune checkpoint inhibitors, genetically engineered T cells, and adoptive cell therapies, have been evaluated both preclinically and clinically [11]. These strategies can be broadly characterized as either stimulating de novo immune responses or boosting existing immunity, according to the baseline immune conditions of patients with HCC; thus, they are considered as potential strategies for treating HCC. However, the efficacy of these treatments has only been observed with limited cancer types, and the therapeutic effect of immunotherapy in advanced HCC is still far from satisfactory [12, 13]. Currently, the effective and efficient treatment of HCC is a primary focus of modern medical research.

The liver is a critical, frontline immune tissue that can detect and clear pathogens; hence, it is susceptible to shifts in inflammation-related cells and cytokines [2]. Maintaining the balance between proinflammatory and anti-inflammatory compounds is critical for immune homeostasis and contributes significantly to overall health. Chronic inflammation and immunotolerance induced by an imbalanced immune system are primary causes of HCC development, recurrence, and metastasis [14]. In patients with HCC, anti-inflammatory compounds, including regulatory *T* (Treg) cells, tumor-associated macrophages (TAMs), myeloid-derived suppressor cells, and liver sinusoidal endothelial cells, express cytokines, such as interleukin- (IL-) 6, IL-10, and transforming growth factor- (TGF-) *β* [15, 16]. Patients with HCC also show low expression of major histocompatibility complexes and high activation of various immune checkpoint inhibitors, including PD-1 and cytotoxic T lymphocyte-associated antigen-4, which suppresses tumor immune responses through subtle regulation [15, 16]. Immune cells as CD4^+^/CD8^+^ T cells, dendritic cells (DCs), and NK cells generate or mediate immune responses to eradicate potential tumor cells. However, the immune responses in the liver are relatively mild compared to those in other tissues, such as the lungs and gastrointestinal tissues, due to the unique immunological state in the liver [16]. Current immunotherapeutic strategies predominately have only one target in the immune system, and combined therapies result in a high incidence of immune-related adverse effects [17]. Hence, the efficacy and efficiency of immunotherapy in liver cancer are relatively low compared to those in other cancers. This may explain why only a limited number of immune-related therapies have been approved by the FDA for treating liver cancer [18].

As antitoxic, cytotoxic, and fitness-enhancing agents, traditional Chinese medicines (TCMs) have long proven effective in alleviating clinical symptoms, improving the quality of life and immune functions, preventing recurrence and metastasis, delaying tumor progression, and prolonging survival in cancer patients [19–21]. In previous decades, mounting clinical and experimental evidence demonstrated the unique therapeutic effects of TCMs and their extracts on HCC. Data from a multicenter, randomized controlled trial conducted by Ling et al. [22] indicate that TCMs helped prevent recurrence after HCC resection. In that study, the median recurrence-free survival in the traditional herbal medicine (THM) group (where cinobufacini injection and jiedu granules were administered) was 46.8 months compared to the median recurrence-free survival of 34.5 months in patients who received only transcatheter arterial chemoembolization (TACE) treatment. The hazard ratio of recurrence between the THM and TACE groups was 0.695 [22]. Another retrospective cohort study showed that administration of jiedu granules added 2.2 months to the median survival time [23]. It could be concluded that jiedu granules, which are composed of four traditional Chinese herbal medicines, *Actinidia valvata*, *Salvia chinensis*, *Cremastra appendiculata,* and the gizzard membrane of *Gallus gallus domesticus*, may improve health and prolong survival in HCC [22, 23]. Su et al. [24] found that the TCM syndrome differentiation in patients with HCC correlated with the degree of *E*-cadherin- or *β*-catenin-dependent cell-cell adhesion and liver function, which bridged the theoretical gap between TCM and modern biology.

The potential mechanisms that underlie the antitumor effects of TCMs include apoptosis, autophagy, and pyroptosis induction in tumor cells; cell cycle arrest and the inhibition of tumor proliferation; impairment of the activity of tumor telomerase; reversing of the multidrug resistance of tumors; regulation of tumor metabolism; and remodeling of the immune microenvironment [19, 25]. Since shifts in immunity have been recognized as the most vital carcinogenetic factor, the immunoregulatory potency of TCMs is gathering increased interest. In a meta-analysis of 12 clinical trials [26], it was concluded that TCM plus TACE compared with TACE alone improved immune responses against HCC in patients and increased the proportions of CD3^+^ and CD4^+^ T cells, as well as the CD4^+^/CD8^+^ T-cell ratio. TCM has been shown to enhance immune responses during HCC treatment and can affect multiple targets to provide health benefits. As mentioned above, single-target treatments are ineffective at reversing the liver-specific immunotolerance that leads to tumor escape. The characteristic multitarget regulation by TCM compounds may provide new insights into effective HCC immunotherapies. Below, we review the immunoregulatory potency of TCMs in the treatment of HCC for the first time and explain how individual TCM drugs and complex TCM formulas remodel the immune environment in various cell- and cytokine-related ways.

### 1.1. Studies on the Immunomodulation by Individual TCM Drugs and Complex Formulas

We searched PubMed and the China National Knowledge Infrastructure databases using the keywords “hepatocellular carcinoma,” “immunology,” and “TCM” to find relevant studies conducted between 2003 and 2020. These works are briefly summarized in Tables 1 and 2. Thirty-six studies, including 6 clinical studies (labeled as “clinical” in tables) and 1 meta-analysis, were conducted in the given timeframe, wherein the immunomodulatory effects of individual TCM drugs (9), TCM compounds (10), or complex formulas (17) were investigated. The underlying mechanisms of treatments that reinforce tumor immunity include increasing the abundances of immunostimulatory cells, such as NK, CD4^+^, and CD8^+^ cells; the upregulation of proinflammatory factors, including IL-2, interferon- (IFN-) *γ*, and tumor necrosis factor- (TNF-) *α*; and the downregulation of several angiogenesis factors and immunosuppressive compounds, such as Treg cells, TAMs, IL-4, IL-10, TGF-*β*, CXCL-8, and CXCL-12 (see Tables 1 and 2).

### 1.2. Individual Drugs and Their Chemical Extracts

#### 1.2.1. TCMs Upregulate Immunostimulatory Factors to Exert Antitumor Effects

HCC is an inflammation-driven disease, with coincident chronic hepatitis and an immunotolerant microenvironment [2]. After infiltrating a tumor, the different cytokines released by immune cells can influence the inflammatory response, inducing either immunostimulation or immunosuppression. Enhanced immunity plays key roles in eradicating residual tumor cells, preventing metastasis and recurrence, and inhibiting oncogenesis in HCC [64–66]. During antitumor immune responses, multiple immune cells are recruited to and congregate around tumor cells. These immune cells interact through a complex series of pathways that ultimately lead to tumor lysis. In addition, released tumor antigens are internalized and presented by antigen-presenting cells, including Kupffer cells, liver sinusoidal endothelial cells, dendritic cells, and hepatocytes. After identifying the activating and costimulatory signals, CD8^+^ T cells quickly proliferate and are primed to kill tumor cells, forming a virtuous cycle for tumor eradication. Recent research has shown that TCM can act as an immune stimulator during HCC treatment [25].

Cinobufagin is a water-soluble extract collected from the exocrine glandular secretions of toads, which is administered in purified form in TCM and is known as *Venenum bufonis* [28]. Cinobufagin has been widely used to treat various kinds of cancer, especially in patients who present with cancer-associated pain [67, 68]. In previous decades, the anticancer and analgesic mechanism of cinobufagin has been widely discussed. Cinobufagin exerts immunological analgesic effects by increasing the activity of the proopiomelanocortin (POMC)/*β*-endorphin (*β*-END)/*μ*-opioid receptor (*μ*-OR) pathway, which is activated by lymphocytes following tumor infiltration [27]. In mice bearing H22 hepatoma cell-derived tumors, intraperitoneal administration of cinobufagin can stimulate the proliferation of CD3^+^, CD4^+^, and CD8^+^ T cells, which in turn increases the expression of the *β*-END, POMC, and *µ*-OR proteins in xenograft tissues and blocks peripheral *µ*-ORs to elicit an analgesic effect. Additionally, the elevated number of immune cells and decreased expression of IL-1*β* lead to enhanced immunity, which improves the survival time of HCC patients [28].

Plant-derived polysaccharides are a class of dietary polysaccharides extracted from natural herbs. They can be consumed orally as food supplements and exert regulatory effects on the functions of both innate and adaptive immunity [69]. The ability of plant-derived polysaccharides to shape the immune response has attracted ever-increasing attention by researchers who wish to explore the antitumor potential of these compounds. *Lycium barbarum* polysaccharide (LBP), extracted from the fruit of the most widely used Chinese herbal medicine, Goji berry, has been reported to have pharmacological and biological activities towards many kinds of hepatitis and HCC [70]. Recently, Chen et al. [30] explored LBP-induced effects on the maturation and differentiation of DC in different HCC environments. They found that LBP increased IL-12p70 and IFN-*γ* secretion in a mixed lymphocyte-reaction system *in vitro*, which stimulated the allogeneic lymphocyte proliferation of DCs. Moreover, LBP augmented nuclear factor- (NF-) *κ*B expression in DCs to different levels in two different tumor microenvironments, and NF-*κ*B expression increased more in the HepG2.2.15 group than in the HepG2 group. This finding indicates that LBP is more effective at enhancing DC maturation in a virus-related HCC environment and that this effect is related to the NF-*κ*B-signaling pathway. He et al. [29] demonstrated that oral LBP administration could increase the numbers of CD4^+^ and CD8^+^ T cells, as well as that of DCs among tumor-infiltrating lymphocytes *in vivo*. Additionally, enhanced B7-1 expression was observed in LBP-treated, H22 tumor-bearing mice, which could provide a costimulatory signal for T-cell activation.

Polysaccharides isolated from *Artemisia apiacea* (HQG) are known for their antitumor effects. HQG treatment can inhibit hepatoma growth in H22 tumor-bearing mice by inducing cancer cell apoptosis and via immunomodulation [71, 72]. HQG treatment increased the numbers of both CD4^+^ and CD8^+^*T* lymphocytes but decreased the ratio of CD4^+^/CD8^+^ T cells, which is associated with enhanced adaptive tumor immunity. HQG treatment also increased the secretion of IFN-*γ* and IL-4, which stimulate Th1- and Th2-mediated immune responses, respectively, in spleen lymphocytes *in vivo* [73].

Corn silk polysaccharides (CSP) have been known to exert antitumor effects for years. Yang et al. [41] found that CSP could inhibit tumor growth and prolong survival times in tumor-bearing mice through an immunostimulatory effect. In H22 tumor-bearing mice, oral administration of CSP resulted in increased body weights; peripheral white blood cell counts; thymus and spleen indices; and serum IL-2, IL-6, and TNF-*α* levels *in vivo*.

The antitumor effects of *Ganoderma lucidum* polysaccharides (GLP) occur through immunomodulation and may represent a unique pathway [32]. It has been found that intragastric GLP administration can restore the immune functions of intestinal mucosae disrupted by 5-fluorouracil (5-FU) in H22 tumor cell-bearing mice, thereby increasing the number of CD8^+^ T cells and elevating the expression of immunoglobulin A, TNF-*α*, and IL-2 in the ileum. The immunosuppressive cytokine IL-10 was also significantly downregulated by GLP. These results indicate that GLP acts on the immune system of the intestinal mucosa to inhibit tumor growth.

Acting as a restorative TCM drug, *Gastrodia elata* Blume has been shown to elicit an immunomodulatory effect [74]. For HCC treatment, *G. elata* can also be used as an auxiliary reagent to relieve symptoms, prevent recurrence, and improve the quality of life [75]. According to phytochemical investigations, this herb contains abundant phenolic compounds, including gastrodin, vanillin, and parishin B. Shu et al. [39] found that, among these chemicals, only gastrodin inhibited H22 tumor cell growth *in vivo*, with low toxicity. The mechanism of its antitumor effect may relate to the enhanced cytotoxic activities of NK and CD8^+^ T cells caused by gastrodin. Intraperitoneal administration of gastrodin specifically upregulated serum IL-2 and IFN-*γ* levels, while decreasing IL-4 levels, thereby transforming the CD4^+^ T-cell profile from being Th2-skewed to being Th1-skewed. Antitumor immunity was further augmented by this shift in the cytokine profile.

Shikonin is the major phytochemical compound isolated from the herbal medicines *Arnebia euchroma* and *Lithospermum erythrorhizon* and is reported to induce apoptosis in various cancer cells, including those of gastric cancer, colon adenocarcinoma, epidermoid carcinoma, leukemia, prostate cancer, and oral squamous cancer [76]. Su et al. [35] found that shikonin inhibited tumor growth and prolonged the survival of H22 and S18 hepatoma tumor-bearing mice, partly due to the recovery of CD3^+^ and CD19^+^ T lymphocyte numbers, enhanced NK cell activity and lymphocyte transformation, and increased IL-2 production *in vivo.* Shikonin can also protect immune system-related organs against tumor-induced damage and restore impaired immune function elicited by combined 5-FU administration.

The rhizome of *Dioscorea bulbifera* L. has been widely used in Asian countries, including China and Bangladesh, since ancient times [77, 78]. The traditional indications of this drug include leprosy, thyroid disorders, and various kinds of cancers. As leprosy and thyroid disorders have become well-managed by modern medicine, modern research has focused more on the antitumor effects of *D. bulbifera*. The main chemical compounds of *D. bulbifera* include diterpenoids, glycosides, steroids, steroidal saponins, and sapogenins [79–82]. Wang et al. [36] screened *D. bulbifera* rhizome extracts obtained with different solvents to investigate the antitumor and immunomodulatory effects of this plant. Among the extracts obtained with four different solvents (water, alcohol, ethyl acetate, and nonethyl acetate), the ethyl acetate extract of *D. bulbifera* increased the weight of the spleen and thymus and increased the total number of leukocytes, lymphocytes, and neutrophils in H22 and S18 tumor-bearing mice. Diosbulbin B is the major bioactive antitumor component of the *D. bulbifera* rhizome, with enhanced immunity and reduced tumor weight being the key effects of this component [36].

Beyond their use in herbal medicine, insects represent important constituents of TCMs used in HCC treatment. According to ancient TCM theory, *Eupolyphaga sinensis* Walker is an effective medicine that can promote blood circulation and remove obstinate blood stasis; obstinate blood stasis resembles the cause and pathological state of HCC [83]. In modern medical research, *E. sinensis* Walker has been shown to enhance immune function, induce cancer cell apoptosis, and inhibit the proliferation of vascular smooth muscle cells to exert antitumor effects [84, 85]. Ge et al. [37] showed that oral administration of an ethanol extract of *E. sinensis* Walker can upregulate Bcl-2/Bax expression and activate caspases-3, thereby mediating H22 tumor cell apoptosis *in vivo*; increased production of the Th1-type cytokines TNF-*α* and IFN-*γ* was also observed, which arrested cancer progression by inducing cancer cell senescence [86]. In previous research focused on adaptive immune tolerance, it was also found that TNF-*α* and IFN-*γ* signaling induce B7–H1 and PD-L1 expression via several pathways and thereby cause immune resistance [87, 88]. The balance between the antitumor and protumor effects of TNF-*α* needs further investigation.

#### 1.2.2. TCMs Downregulate Immunosuppressive Factors to Enhance Tumor Immunity

As an organ that is constantly susceptible to immunotolerance, the liver maintains an immunosuppressive microenvironment, which facilitates tumor evasion from immunosurveillance. Therefore, it is hard for therapeutic regimens to eradicate tumors in patients with advanced HCC [2]. Regarding virus-mediated HCC, various immune-tolerogenic cells and cytokines are generated by malignant cells and stromata associated with chronic inflammation, which facilitates a rapid and subtle counteraction against the tumor immunity acquired in HCC [16]. In the HCC microenvironment, the most common immunosuppressive cells include TAMs, tumor-associated neutrophils, marrow-derived suppressor cells, and Treg cells. These cells congregate within or around the edges of tumors and express immunosuppressive cytokines, chemokines, or cell-surface ligands, including C-X-C motif ligand (CXCL)17, CXCL18, CXCL22, vascular endothelial growth factor (VEGF), TGF-*β*, IL-10, and PD-L1; this inhibits or effectively inactivates cytotoxic immune cells and promotes tumor growth [2, 13, 15, 17, 18]. In this case, inhibiting negative regulation and reversing the immunosuppressive environment may help restore proper functioning of the immunosurveillance system and antitumor immunity. In TCM, various medical compounds are in use that can potently stimulate immune responses and reverse the immunotolerance of the liver.

*Astragalus* polysaccharides (APs) are the main phytochemical compounds isolated from the traditional herbal medicine *Astragalus membranaceus*. The immunomodulatory activities of AP have been widely reported in previous studies [89, 90]. APs can stimulate macrophage-induced 4T1 tumor cell apoptosis without any direct cytotoxic effects [90]. AP can also alleviate the immune lesions caused by 5-FU in combined therapy [91]. Treg cells are an exceptional subset of CD4^+^ T lymphocytes that express CD25 and FOXP3 [65]. Under normal circumstances, Treg cells protect the body from aberrant immunity that is overactivated by gut microbiota antigens, food antigens, and other autoantigens. However, in the tumor microenvironment, the high secretion of IL-10 and TGF-*β* by tumor-infiltrating Treg cells can facilitate tumor growth and invasion [92]. Treg cells that highly express CTLA-4 inhibit the cytotoxic function of CD8^+^ T cells by binding to their surface B7 receptors. In general, a high proportion of Treg cells is associated with tumor progression and serves as a poor prognostic indicator in patients with HCC [93, 94]. Li et al. [38] found that, in HCC patients, oral administration of APs reduced FOXP3 expression in the local HCC environment, thus inhibiting Treg cells. APs can also block the Treg-recruiting ability of SDF-1 via the CXCR4/CXCL12 pathway and further inhibit Treg migration *in vitro*.

*Radix Glycyrrhizae* polysaccharide (RGP) is a major bioactive compound isolated from another important TCM plant, *R. Glycyrrhizae*. RGP can modulate the immune function of macrophages and reduce oxidative stress within tumors [95]; moreover, RGP can inhibit Treg cell activity in H22 tumor-bearing mice [33]. According to their profiles of secreted cytokines, CD4^+^ T cells can be divided into Th1 and Th2 cells. Th1 CD4^+^ T cells are proinflammatory, producing high levels of IL-2, IFN-*γ*, IFN-*α*, and TNF-*β* to activate CD8^+^ T and NK cells, which enhance innate and adaptive immune responses, ultimately exerting an antitumor effect. Th2 CD4^+^ T cells produce IL-4, IL-10, and TGF-*β*, which may boost tumor progression. The ratio of Th1 and Th2 cells is another immune biomarker used to prognose tumor progression aside from tumor-infiltrating Treg cells; a lower Th1/Th2 cell ratio indicates a poor prognosis. He et al. [33] found that administering RGP increased IL-2 and IL-12p70 production, while decreasing serum IL-10 and TGF-*β* levels in H22 tumor-bearing mice, in accordance with upregulated Th1 and downregulated Th2.

Glycyrrhizin is another bioactive compound isolated from *R. Glycyrrhizae*. A recent study has reported that the strong antitumor effects of glycyrrhizin are a result of autophagy induction in HCC [96]. In the liver stroma, the largest population of resident macrophages (Kupffer cells) accounts for approximately 90% of all tissue-resident macrophages [97]. Kupffer cells play a key role in detecting and clearing pathogens, including viruses, bacteria, and other exogenous antigens, relying upon complex interactions between Kupffer cells and microbicidal neutrophils [97, 98]. However, Kupffer cells can also be responsible for immune hyporesponsiveness and confer immunotolerance in antitumor immunity. With PD-L1 expressed on the surface of Kupffer cells, the tumor antigen cross-presentation by Kupffer cells is more likely to induce immunotolerance than activation of naïve CD8^+^ T cells, due to the inhibitory signal initiated by PD1/PD-L1 binding. Macrophages can be divided into proinflammatory M1 and anti-inflammatory M2 cells, based on their cytokine profiles and immune functions [2, 99, 100]. TAMs are mainly composed of M2 cells, which are stimulated by cytokines, such as IL-4 and IL-13, that are largely released from Th2 lymphocytes. In HCC, TAM and Kupffer cells are characteristic immunosuppressive factors that mediate tumor evasion; the abundance of these cells is a negative biomarker for HCC prognosis. Wang et al. [43] found that the intravenous administration of glycyrrhizin regulated the Bax/Bcl-2 ratio and caspase-3 activity, leading to Kupffer cell inactivation. Tumor immunity is consequently enhanced by the attenuation of the suppressive function of Kupffer cells and TAMs. The results of that study also showed that injection of doxorubicin-alginate nanogel particles combined with glycyrrhizin was more efficacious than the injection of nanogel particles alone, as nanogel particles were retained in the liver due to the reduced phagocytosis of macrophages *in vivo*.

Triterpenoid saponins are chemicals isolated from the herbal medicine *Anemone flaccida* Fr. Schmidt; these chemicals inhibit tumors by inducing apoptosis *in vitro* [101, 102]. Han et al. [44] found that oral administration of triterpenoid saponins in H22 tumor-bearing mice enhanced both innate and adaptive immunity, which resulted from a decreased number of Treg cells and an increased number of B cells, NK cells, and CD3^+^/CD28^+^ T cells in the spleen and tumor tissues. Triterpenoid saponins can also reverse aberrant cell-signaling pathways, which are involved in the formation of immune lesions and, therefore, tumor evasion. Certain proinflammatory cytokines, such as TGF-*β*, VEGF, and cyclooxygenase (COX)-2, can induce tumor progression in different ways [103]. COX-2 is highly expressed in the presence of excessive local levels of TNF-*α* due to the activation of NF-*κ*B-signaling pathways in malignant cells [104]. Triterpenoid saponins significantly downregulate NF-*κ*B expression, leading to reduced secretion of local COX-2 and its synthase (membrane-associated prostaglandin E synthase-1), thereby reversing the immunosuppressive cytokine profile [44]. In terms of the immune checkpoint-signaling pathway involved in HCC immunotolerance, the janus kinase (JNK), signal transducer and activator of transcription (STAT), and mitogen-activated protein kinase (MAPK) signals are key for PD1/PD-L1 activation. It has also been found that triterpenoid saponins diminish the activation of JNK/STAT and MAPK signaling *in vivo*, thus blocking PD1/PD-L1 activation in HCC [44]. Generally, triterpenoid saponins can influence multiple signaling cascades involved in HCC tumorigenesis and tumor immunity.

### 1.3. TCM Formulas and Mixed Compounds

TCM formulas are made up of a set of traditional medicines with homogeneous properties based on the principles of prescription formulation and basic TCM theory. Compared to individual drugs, TCM formulas can exert more synergistic and comprehensive effects on the body. For HCC treatment, various TCM formulas are capable of addressing complications, reducing the adverse effect of certain drugs, strengthening the treatment efficiency, and achieving durable efficacy. Hence, formulas are applied more frequently in routine TCM prescriptions that are individual drugs.

#### 1.3.1. Formulas Upregulate Immunostimulatory Factors to Exert Antitumor Effects

Similar to individual drugs, some TCM formulas directly strengthen immunity, leading to inhibited tumor progression. The Ciji-Hua'ai-Baosheng decoction is a TCM formula introduced in recent years to treat cancer patients and contains five main compounds: *Radix Salviae* Miltiorrhizae, *Codonopsis Radix*, *Poria*, *Bulbus Fritillariae* Ussuriensis, and *Concha Ostreae* [56, 57]. Ciji-Hua'ai-Baosheng granules can prolong the survival of HCC-bearing mice receiving chemotherapy [105]. Xi et al. [58] demonstrated that the intragastric administration of Ciji-Hua'ai-Baosheng granules improved innate immunity by increasing the weight and indices of the thymus and spleen, thereby inhibiting ectopic H22 tumor growth in mice. Ciji-Hua'ai-Baosheng granules also upregulated the expression of IL-2, IFN-*γ*, and TNF-*α* in both serum and tumor tissues *in vivo*, which can help resist immune-related injuries caused by chemotherapy. Decreased levels of IL-6 (which counters the apoptosis induced by chemotherapy) and high IL-6 levels (which negatively impact cancer prognosis) have also been observed [106]. Ultra-high-performance liquid chromatography identified 11 bioactive compounds in this decoction that possess immunostimulating properties, including hesperidin, spinosin, naringin, salvianolic acid c, lithospermic acid, vitexin, baicalin, caffeic acid, salvianolic acid b, nobiletin, and cryptotanshinone [58].

The immune-tolerant characteristics and specific anatomical structure of the liver make it a site where primary tumors commonly develop and tumor metastasis from other organs frequently occurs [2]. The most common occurrence is the metastasis of primary colorectal cancer to the liver. Lee et al. [46] found that oral administration of Bojung-bangam-tang, a new herbal prescription, could increase the secretion of Th1-like cytokines (IL-12 and IFN-*γ*) by macrophages and decrease Th2-like cytokine (IL-4) production. IL-12 is widely known for its antimetastatic ability to activate Th1 cell-dominant immunity, such as by enhancing the cytotoxic function of NK cells, increasing IFN-*γ* production, and stimulating and licensing CD8^+^ T cells, which are involved in both innate and adaptive immunity [107]. IFN-*γ* is another immunostimulating cytokine that plays a pivotal role in tumor detection and clearance. The immunomodulatory effect of Bojung-bangam-tang leads to a reduced rate of metastasis of colorectal cancer to the liver.

The modified Si-Jun-Zi decoction is a widely applied TCM formula dating back to ancient times, which contains 4 herbs: *Codonopsis pilosula*, *Poria*, *Atractylodis Macrocephalae*, and *Glycyrrhizae Radix*. In TCM theory, Si-Jun-Zi decoction is a Qi-enhancer in the body. In modern medical research, it has been claimed that the decoction effectively reinforces immune functions in patients with chronic inflammation [108]. Recently, it has been shown that the intragastric administration of a modified Si-Jun-Zi decoction can inhibit colorectal cancer liver metastasis and raise survival rates, both alone and when combined with 5-FU treatment in a mouse model of orthotropic colon cancer [60]. In a colon tumor-bearing mouse group treated with a modified Si-Jun-Zi decoction, the levels of serum cytokines, such as IL-1, IL-3, GM-CSF, and IFN-*γ*, increased significantly, stimulated the proliferation of M1 macrophages, and improved innate immunity. In H22 hepatoma tumor-bearing mice, intragastric administration of a modified Si-Jun-Zi decoction increased the thymus and spleen indices in a dose-dependent manner [109]. The modified decoction also increased the serum IL-2 and TNF-*α* levels. Upregulated Bax and caspase-3 expression, alongside Bcl-2 downregulation, which are associated with cancer cell apoptosis, have also been observed following administration of a modified Si-Jun-Zi decoction.

In an experimental formula, the immunomodulatory effect of Shiquan-Yuzhen decoction was investigated [53]. Intragastric administration of Shiquan-Yuzhen decoction in H22 tumor-bearing mice increased the thymus and spleen indices; upregulated the expression of CD3, CD4, CD8, and TNF-*α* receptors in lymphocytes; and decreased the expression of IL-2 and IFN-*β*. Both innate and adaptive immunity were reinforced following administration of this formula. It was also found that Fuzheng-Qingjie granules (which reduce heat and clear toxins according to basic TCM theory) inhibited HCC growth and boosted immune function. Fuzheng-Qingjie granules can not only induce mitochondria-mediated apoptosis in HepG2 hepatoma cells *in vitro* but also increase the number of CD4^+^ T lymphocytes, NK cells, and ratio of CD4^+^/CD8^+^ T cells in the peripheral blood of H22 subcutaneous tumor-bearing mice [54]. Upregulated serum IL-2 and TNF-*α* levels and increased CD69 expression on the surface of tumor cells were also observed, which correlated with enhanced adaptive immunity. A meta-analysis that included 29 clinical trials and 2,488 patients with advanced HCC was performed to investigate the efficacy and safety of a Chinese patent medicine—Jinlong capsules—in treating advanced HCC [61]. Jinlong capsules increased the ratio of CD4^+^ to CD8^+^ T cells and the percentages of NK, CD3^+^, and CD4^+^ cells in patients with advanced HCC, indicating that it reinforced antitumor immunity. In a clinic trial involving 48 patients with primary liver cancer [62], Jinlong capsules increased the levels of IL-2 in patients while inhibiting sIL-2R levels and improving lymphocyte functions in patients after TACE treatment.

Fuzheng-Yiliu decoction is a TCM formula purported to strengthen vital energy and enhance the body's resistance to cancer, following a major principle of Fuzheng-Guben formulas. It has been reported that treatment with a combined Fuzheng-Yiliu decoction can improve the efficacy and alleviate the adverse effects of chemotherapy in the treatment of malignant gastrointestinal tumors [110]. Cao et al. [50] presented evidence demonstrating that the intragastric administration of the Fuzheng-Yiliu decoction increased the serum levels of IL-2 and TNF-*α* and elevated the thymus index in H22 tumor-bearing mice. When combined with 5-FU, administration of the Fuzheng-Yiliu decoction reversed immunosuppression, increasing the total number of WBCs and lymphocytes that were diminished by 5-FU. Increased percentages of CD3^+^, CD4^+^ T cells, and NK cells in the peripheral blood were observed in another study [48]. A clinical study exploring the immunomodulatory effect of the Fuzheng-Yiliu decoction on HCC revealed that the CD4^+^/CD8^+^ T-cell ratio increased in the group administered TCM [49].

#### 1.3.2. Formulas Downregulate Immunosuppressive Factors to Enhance Tumor Immunity

Among all immunosuppressive signals related to HCC tumor immunity, the NF-*κ*B/IL-6/STAT3 inflammatory axis is crucial for promoting tumor proliferation and evasion, with protumor cytokines being highly upregulated by this pathway. The immunosuppressive factors involved in the NF-*κ*B/IL-6/STAT3 axis include IL-6, IL-10, IL-1*β*, TGF-*β*, and VEGF [111, 112]. In this signaling cascade, NF-*κ*B activation stimulates the production of IL-6, which is a proinflammatory cytokine that is critical for creating a suitable environment for tumor growth. STAT3 signaling activated by IL-6 can in turn stimulate NF-*κ*B, thereby forming a vicious cycle [113]. Lu et al. [59] demonstrated that the TCM formula NHE-06 inhibited the NF-*κ*B/IL-6/STAT3 inflammatory signaling in both HCC and macrophage cell lines, *in vitro*. NHE-06 administration decreased IL-6 levels and downregulated IL-1*β* and COX-2 expression in mice bearing subcutaneous Hepa1-6 tumors, which is indicative of inactivated NF-*κ*B/IL-6/STAT3 signaling and restored tumor immunity without direct tumor cytotoxicity.

Thymic stromal lymphopoietin (TSLP) is an immunosuppressive IL-17-like cytokine that promotes Th2-dominant immune responses and facilitates tumor progression. TSLP signaling can induce VEGF activation through the JAK/STAT pathway and thereupon promote angiogenesis in the tumor microenvironment [114–116]. Angiogenesis promotes immunosuppression and causes poor prognosis in patients with HCC. Yu-Ping-Feng San is a classic TCM formula that tonifies Qi levels in the lungs and spleen according to TCM theory; this correlates with enhanced disease resistance [117]. The Yu-Ping-Feng San decoction is composed of *Astragali Radix*, *Atractylodis Macrocephalae* Rhizoma, and *Saposhnikoviae Radix*. Yuan et al. [63] found that the intragastric administration of the Yu-Ping-Feng San decoction downregulated tumor TSLP and VEGF levels, while inhibiting angiogenesis in mice with orthotopically transplanted Hepa1-6 cells. This effect was confirmed by the inhibition of the TSLP/STAT3 signaling pathway after Yu-Ping-Feng San treatment in vitro.

Consuming the TCM decoction Songyou Yin coupled with swimming exercises has already shown beneficial effects in terms of tumor growth inhibition and metastasis in patients with HCC [52]. Using subcutaneous Hep1-6 tumor-bearing and experimental lung metastasis mouse models, Zhang et al. [52] found that the oral administration of Songyou Yin suppressed tumor growth and lung metastasis by increasing CD4^+^ T cells in the peripheral blood and reducing the percentage of Treg cells in blood, spleen, and tumor tissues. Reduced serum TGF-*β* levels also promoted CD4^+^ and CD8^+^ T-cell differentiation.

## 2. Conclusions

TCMs play a key role in cancer treatment and immune regulation. In HCC, the unique immunotolerant environment tends to lead to tumor evasion, progression, and metastasis, which can be reversed by the application of TCM [118]. Mounting evidence has indicated that TCMs enhance tumor immunity both through a direct immunostimulatory effect and by removing immunosuppressive factors, including cells, cytokines, and other proteins. CD4^+^ (primarily Th1) and CD8^+^ (primarily cytotoxic T cell) lymphocytes are the main components of antitumor adaptive immunity and are also the main targets against which TCMs exert their invigorating effects. TCMs upregulate several immune-stimulatory cytokines, like IL-1, IL-2, TNF-*α*, and IFN-*γ*, to remodel the adaptive immune system. DC and NK cells themselves act as powerful scavengers in the innate immune system due to their tumor-clearing effects; moreover, the abundant levels of cytokines secreted by these cells also greatly influence the adaptive immune system. TCMs can regulate the abundance and activity of DC and NK cells to modulate tumor immunity. In terms of immunosuppressive factors, Treg cells (primarily CD4^+^ FOXP3^+^ T lymphocytes) and TAMs (primarily M2 macrophages) are clusters of cells that impede tumor immunity via complex regulation. Cytokines, like IL-10, IL4, IL-6, and TGF-*β*, released by these cells can inhibit both innate and adaptive immunity and can also directly promote tumor progression. Destruction of the immune balance is a primary cause of oncogenesis, tumor evasion, and metastasis. Restoring the immune balance and regaining vigorous immunity are the main aim for exerting antitumor effects in HCC through the use of various immunotherapies.

Compared with western medicine, TCMs offer advantages in HCC treatment. The first and foremost advantage is economic. Compared with the price of sorafenib (which currently costs up to $7,223/month), the cost of TCM treatment is relatively low. Additionally, the lower adverse effects and multitarget regulation characteristics are strengths of TCM in terms of comprehensive treatment of HCC. In the early stage of tumor development, TCMs can work as a therapeutic regimen to alleviate protumor factors, such as endocrine disorders, nonresolving inflammation, and biocarcinogens. However, in later stages, TCMs can work as an adjuvant therapy to improve survival, lower drug-related adverse effects, and improve patients' quality of life. Based on these findings, we advocate applying TCM during all stages of HCC development.

**Figure 1:**
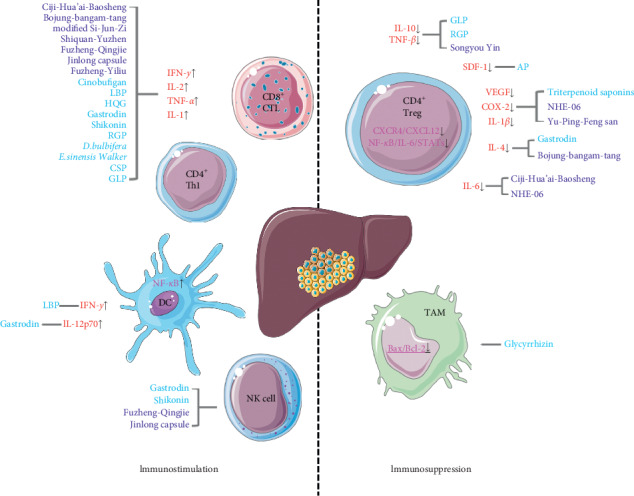
TCMs remodel tumor immunity via immunostimulation and reversing immunosuppression.

Herein, we have summarized immune-related pathways that are targeted by TCMs for treating HCC. The major drugs and immune components regulated by these drugs are indicated in Figure 1. Based on the findings presented in this review, it can be hypothesized that utilizing individual TCM drugs and combined therapy with TCM formulas could be effective in HCC eradication through immunoregulation.

## Figures and Tables

**Table 1:** Individual drugs or their chemical compounds enhance tumor immunity.

Year	Drug	Source(s) of drug	The mode of administration	Change(s) of signal pathway involved	Change(s) of immune compounds	Ref.
2003; 2017	Cinobufagin	*Venenum bufonis*	Intravenous (clinical); intraperitoneal administration	Proopiomelanocortin/*β*-endorphin/*μ*-opioid receptor pathway	CD3+, CD4+, and CD8+ T cells ↑; IL-1*β* ↓	[27, 28]
2005; 2012	*Lycium barbarum* polysaccharide	Goji berry	*In vitro*; oral	NF-*κ*b pathway↑	IL-12p70 and IFN-*γ* ↑; DC ↑; CD4+ and CD8+ T cells ↑	[29, 30]
2007	Standardized silymarin extract	Milk thistle	*In vitro*	NF-*κβ* pathway ↓; JAK-STAT pathway ↑	TNF-*α* ↓; CXCL8 transcription ↓; IFN-*α* ↑	[31]
2009	*Ganoderma lucidum* polysaccharides	*Ganoderma lucidum*	Intragastric	None	CD8+ T cells ↑; immunoglobulin A ↑; TNF-*α* ↑; IL-2 ↑; IL-10 ↓	[32]
2011	*Radix Glycyrrhizae* polysaccharide	*Radix Glycyrrhizae*	Intraperitoneal; subcutaneous	Th1-skew immune response ↑; Th2-skew immune response ↓	IL-2 and IL-12p70 ↑; IL-10 and TGF-*β* ↓	[33]
2011	*Strongylocentrotus nudus* eggs polysaccharide	Sea urchins	Intraperitoneal	NFAT pathway ↑; MAPK/ERK pathway ↑	CD4+ and CD8+ T lymphocytes ↑; CTL cytotoxicity ↑; serum IgG, IgM, and IgA antibody ↑; IL-2 and TNF-*α* ↑; Erk1/2 ↑	[34]
2012	Shikonin	*Arnebia euchroma*; *Lithospermum erythrorhizon*	By gavage	None	NK, CD3+, and CD19+ T cells ↑; IL-2 ↑	[35]
2012	*Dioscorea bulbifera* L. extract	*Dioscorea bulbifera* L.	Intragastric	None	Spleen and thymus weight ↑; total leukocytes, lymphocytes, and neutrophils ↑	[36]
2012	*Eupolyphaga sinensis* Walker extract	*Eupolyphaga sinensis* Walker	Intragastric	Th1-skew immune response ↑	TNF-*α* and IFN-*γ* ↑	[37]
2012	*Astragalus* polysaccharides	*Astragalus membranaceus*	Oral (clinical)	CXCR4/CXCL12 pathway↓	FOXP3+ Treg ↓; SDF-1 ↓	[38]
2013	Gastrodin	*Gastrodia elata* Blume	Intraperitoneal	Th1-skew immune response ↑; Th2-skew immune response ↓	NK and CD8+ T cells ↑; serum IL-2 and IFN-*γ* ↑; serum IL-4 ↓	[39]
2013	Xiaoaiping	*Marsdenia tenacissima*	Intravenous injection (clinical)	None	Peripheral CD3+ and CD4+ T cells ↑	[40]
2014	Corn silk polysaccharides	Corn	Oral	None	Peripheral WBC count ↑; thymus and spleen index ↑; serum IL-2, IL-6, and TNF-*α* ↑	[41]
2014	Ginsenoside Rg3	Ginseng	Intraperitoneal	None	IL-2 and IFN-*γ* ↑	[42]
2019	Glycyrrhizin	*Radix Glycyrrhizae*	Intravenous	Bax/Bcl-2 ↑	Kupffer cells and TAM ↓	[43]
2019	Triterpenoid saponins	*Anemone flaccida* Fr. Schmidt	Oral	NF-*κβ* pathway ↓; JNK/STAT pathway ↓; PD-1/PD-L1 pathway	B cells, NK cells, and CD3+/CD28+ T cells ↑; FOXP3+ Tregs ↓; TNF-*α*, TGF-*β*, COX-2, and mPGES1 ↓; ERK1/2 and p38 ↓	[44]
2019	Icaritin	*Epimedii herba*	Oral (clinical)	M1-type macrophages activation↑; M2-type macrophage activation ↓	Total neutrophils ↓; total lymphocytes ↑; MDSC populations ↓; serum IL-6, IL-7, IL-8, IL-10, and IL-15 ↓; serum IFN-*γ* ↑	[45]

NF-*κ*B: nuclear factor-*κ*B; IL: interleukin; IFN: interferon; JAK-STAT: janus kinase-signal transducer and activator of transcription; TNF: tumor necrosis factor; CXCL : C-X-C motif ligand; CXCR : C-X-C motif receptor; TGF-*β*: transforming growth factor-*β*; NFAT: nuclear factor of activated T cells; MAPK/ERK: mitogen-activated protein kinase/extracellular regulated protein kinases; CTL: cytotoxic T lymphocyte; Ig: immunoglobulin; NK: natural killer; CD: cluster of differentiation; Treg: regulatory T cell; SDF-1: stromal cell-derived factor-1; Bax/Bcl : BCL2-associated X/B-cell lymphoma; TAM: tumor-associated macrophage; JNK : Jun *N*-terminal kinase; PD: programmed cell death; COX: cyclooxygenase; mPGES: membrane associated prostaglandin E synthase; MDSC: myeloid-derived suppressor cells.

**Table 2:** TCM formula or mixed compounds enhance tumor immunity.

Year	Formula	Compounds contained in formula	The mode of administration	Change(s) of signal pathway involved	Change(s) of immune compounds	Ref.
2003	Bojung-bangam-tang	*Agaricus* mushroom, *Atractylodis Macrocephalae* Rhizoma, *Ginseng Radix*, *Polyporus*, *Citri Pericarpium*, *Astragali Radix*, *Dioscoreae Rhizoma*, *Angelicae gigantis Radix*, and *Glycyrrhizae Radix*	Oral	Th1-skew immune response ↑; Th2-skew immune response ↓	IL-12 and IFN-*γ* ↑; IL-4 ↓	[46]
2008	Xiaochaihu decoction	*Radix bupleuri*, *Scutellaria baicalensis*, *Pinellia ternata*, Chinese-date, and ginger	By gavage	None	NK and total lymphocytes ↑; spleen index ↑; thymus gland index ↓; IL-2 ↑	[47]
2011; 2012; 2013	Fuzheng-Yiliu decoction	*Astragalus membranaceus*, *Angelica sinensis*, *Curcuma zedoaria*, and *Patrinia heterophylla*	Intragastric (clinical)	None	Serum IL-2 and TNF-*α* ↑; thymus index ↑; total WBC and lymphocytes ↑	[48–50]
2013	Huangqi-Sijunzi decoction	*Astragalus membranaceus*, *Codonopsis pilosula*, *Rhizoma Atractylodis*, *Poria cocos*, and *Glycyrrhiza*	By gavage	None	NK ↑; CD4+/CD8+ T-cell ratio ↑; FOXP3+ Treg ↓	[51]
2016	Songyou yin	*Astragalus membranaceus*, *Salvia miltiorrhiza*, *Lycium barbarum* L., turtle shell, and hawthorn	Oral	None	CD4+ T cells ↑; FOXP3+ Treg ↓; serum TGF-*β* ↓	[52]
2017	Shiquan-Yuzhen decoction	*Panax ginseng*, *Astragalus membranaceus*, *Rhizoma Dioscoreae*, *Anemarrhena asphodeloides*, *Radix scrophulariae*, *Salvia miltiorrhiza*, *Rhizoma sparganii*, *Curcuma zedoaria*, fossil fragments, and concha ostreae	Intragastric	None	Thymus and spleen indices ↑; lymphocytes CD3, CD4, CD8, and TNF-*α* receptors ↑; IL-2 and IFN-*β* ↓	[53]
2017	Fuzheng-Qingjie decoction	*Astragalus membranaceus*, *Ligustrum lucidum*, *Ganoderma lucidum*, *Rhizoma Dioscorea*, *Hedyotis diffusa* Willd, and *Prunella vulgaris*	By gavage	None	Peripheral CD4+ T cells and NK cells ↑; CD4+/CD8+ T cells ratio ↑; serum IL-2 and TNF-*α* ↑; CD69 on tumor cells ↑	[54]
2017	JC-001	*Bupleurum chinense* DC., *Gentiana scabra* Bunge, *Rheum palmatum* L., *Clematis montana* Buch., *Carthamus tinctorius* L., *Prunus persica* (L.) Batsch, *Angelica dahurica* (Fisch ex Hoffm) Benth et Hook f, *Siegesbeckia orientalis* L., *Glycyrrhiza uralensis* Fisch, and *Solanum incanum* L.	*In vitro*; oral	None	Th1, Th2 ↑; Th17, Treg ↓; IL-6, IL-10, and TNF-*α* ↑; IFN-*γ*, IL-17, IL-3, and IL-1*α* ↓	[55]
2018; 2019	Ciji-Hua'ai-baosheng decoction	*Radix Salviae* Miltiorrhizae, *Radix Codonopsis*, *Poria*, *Bulbus Fritillariae* Ussuriensis, and *Concha Ostreae*	Intragastric	None	Thymus and spleen weight and indices ↑; IL-2, IFN-*γ*, and TNF-*α* ↑; IL-6 ↓	[56–58]
2018	NHE-06	*Ficus carica*, *Folium ginkgo*, *Angelica sinensis*, *Lonicera japonica*, *Pseudo-ginseng*, and *Taraxacum mongolicum*	Not given	NF-*κ*B/IL-6/STAT3 ↓	IL-6, IL-1*β*, and TNF-*α* ↓; PTGS2 and COX-2 ↓	[59]
2019	Modified Si-Jun-Zi decoction	*Codonopsis pilosula*, *Poria*, *Atractylodis Macrocephalae*, and *Glycyrrhizae Radix*	Intragastric	Bax/Bcl-2 ↑	IL-1, IL-2, IL-3, TNF-*α*, GM-CSF, and IFN-*γ* ↑; thymus and spleen indices ↑; Bax and caspase-3 ↑; Bcl-2 ↓	[60]
2008; 2019	Jinlong capsule	*Gekko*, *Bungarus*, and *Agkistrodon*	Oral (meta-analysis; clinical)	None	NK, CD3+, and CD4+ cells ↑; CD4+/CD8+ T cells ratio ↑; IL-2 ↑	[61, 62]
2019	Yu-Ping-Feng san	*Astragali Radix*, *Atractylodis Macrocephalae* Rhizoma, and *Saposhnikoviae Radix*	Intragastric	TSLP/STAT3 ↓	TSLP ↓; VEGF ↓	[63]

IL: interleukin; IFN: interferon; NK: natural killer; TNF: tumor necrosis factor; WBC: white blood cell; CD: cluster of differentiation; Treg: regulatory T cell; TGF-*β*: transforming growth factor-*β*; NF-*κ*B: nuclear factor-*κ*B; STAT: signal transducer and activator of transcription; PTGS: prostaglandin-endoperoxide synthase; COX: cyclooxygenase; GM-CSF: granulocyte-macrophage colony stimulating factor; Bax/Bcl : BCL2-associated X/B-cell lymphoma; TSLP: thymic stromal lymphopoietin; VEGF: vascular endothelial growth factor.

## References

[1] Chen W., Zheng R., Baade P. D. (2016). Cancer statistics in China, 2015. *CA: A Cancer Journal for Clinicians*.

[2] Kubes P., Jenne C. (2018). Immune responses in the liver. *Annual Review of Immunology*.

[3] Nault J. C., Villanueva A. (2020). Biomarkers for hepatobiliary cancers. *Hepatology*.

[4] Chew S. A., Moscato S., George S., Azimi B., Danti S. (2019). Liver cancer: current and future trends using biomaterials. *Cancers (Basel)*.

[5] Yu S. J. (2016). A concise review of updated guidelines regarding the management of hepatocellular carcinoma around the world: 2010-2016. *Clinical and Molecular Hepatology*.

[6] Bruix J., Qin S., Merle P. (2017). Regorafenib for patients with hepatocellular carcinoma who progressed on sorafenib treatment (resorce): a randomised, double-blind, placebo-controlled, phase 3 trial. *The Lancet*.

[7] Ranieri G., Marech I., Lorusso V. (2014). Molecular targeting agents associated with transarterial chemoembolization or radiofrequency ablation in hepatocarcinoma treatment. *World Journal of Gastroenterology*.

[8] Longo V., Gnoni A., Gardini A. C. (2017). Immunotherapeutic approaches for hepatocellular carcinoma. *Oncotarget*.

[9] El-Khoueiry A. B., Sangro B., Yau T. (2017). Nivolumab in patients with advanced hepatocellular carcinoma (checkmate 040): an open-label, non-comparative, phase 1/2 dose escalation and expansion trial. *The Lancet*.

[10] Xu W., Liu K., Chen M. (2019). Immunotherapy for hepatocellular carcinoma: recent advances and future perspectives. *Therapeutic Advances in Medical Oncology*.

[11] Greten T. F., Lai C. W., Li G., Staveley-O’Carroll K. F. (2019). Targeted and immune-based therapies for hepatocellular carcinoma. *Gastroenterology*.

[12] Fu Y., Liu S., Zeng S., Shen H. (2019). From bench to bed: the tumor immune microenvironment and current immunotherapeutic strategies for hepatocellular carcinoma. *Journal of Experimental & Clinical Cancer Research*.

[13] Johnston M. P., Khakoo S. I. (2019). Immunotherapy for hepatocellular carcinoma: current and future. *World Journal of Gastroenterology*.

[14] Wang S., Liu Y., Feng Y. (2019). A review on curability of cancers: more efforts for novel therapeutic options are needed. *Cancers (Basel)*.

[15] De Martin E., Michot J.-M., Papouin B. (2018). Characterization of liver injury induced by cancer immunotherapy using immune checkpoint inhibitors. *Journal of Hepatology*.

[16] Makarova-Rusher O. V., Medina-Echeverz J., Duffy A. G., Greten T. F. (2015). The yin and yang of evasion and immune activation in HCC. *Journal of Hepatology*.

[17] Faivre S., Rimassa L., Finn R. S. (2020). Molecular therapies for HCC: looking outside the box. *Journal of Hepatology*.

[18] Hilmi M., Vienot A., Rousseau B., Neuzillet C. (2019). Immune therapy for liver cancers. *Cancers (Basel)*.

[19] Wang Y., Zhang Q., Chen Y. (2020). Antitumor effects of immunity-enhancing traditional Chinese medicine. *Biomedicine & Pharmacotherapy*.

[20] Zhai B., Zhang N., Han X. (2019). Molecular targets of *β*-elemene, a herbal extract used in traditional Chinese medicine, and its potential role in cancer therapy: a review. *Biomedicine & Pharmacotherapy*.

[21] Liao Y.-H., Lin C.-C., Lai H.-C., Chiang J.-H., Lin J.-G., Li T.-C. (2015). Adjunctive traditional Chinese medicine therapy improves survival of liver cancer patients. *Liver International*.

[22] Zhai X.-f., Chen Z., Li B. (2013). Traditional herbal medicine in preventing recurrence after resection of small hepatocellular carcinoma: a multicenter randomized controlled trial. *Journal of Integrative Medicine*.

[23] Chen L. Y., Zhai X. F., Chen Z. (2017). Jie-du granule preparation for the treatment of advanced hepatocellular carcinoma: a retrospective cohort study of 177 patients. *Oncotarget*.

[24] Xiaokang S., Yusheng W., Yonghao L., Xianwei Y., Yuyun L., Jianan C. (2009). Relationship between TCM syndrome differentiation of liver cancer and adhesion degree of cancer cell. *Zhongguo Zhongxiyi Jiehe Waike Zazhi*.

[25] Lin W.-f., Lu J.-y., Cheng B.-b., Ling C.-q. (2017). Progress in research on the effects of traditional chinese medicine on the tumor microenvironment. *Journal of Integrative Medicine*.

[26] Meng M.-B., Wen Q.-L., Cui Y.-L., She B., Zhang R.-M. (2011). Meta-analysis: traditional Chinese medicine for improving immune response in patients with unresectable hepatocellular carcinoma after transcatheter arterial chemoembolization. *Explore*.

[27] Chen T., Yuan S., Wan X.-n. (2017). Chinese herb cinobufagin-reduced cancer pain is associated with increased peripheral opioids by invaded CD3/4/8 lymphocytes. *Oncotarget*.

[28] Chen Z., Zhai X. F., Su Y. H. (2003). Clinical observation of cinobufacini injection used to treat moderate and advanced primary liver cancer. *Journal of Chinese Integrative Medicine*.

[29] He Y.-L., Ying Y., Xu Y. L., Su J. F., Luo H., Wang H. F. (2005). Effects of Lycium barbarum polysaccharide on tumor microenvironment T-lymphocyte subsets and dendritic cells in H22-bearing mice. *Journal of Chinese Integrative Medicine*.

[30] Chen J.-R., Li E.-Q., Dai C.-Q. (2012). The inducible effect of LBP on maturation of dendritic cells and the related immune signaling pathways in hepatocellular carcinoma (HCC). *Current Drug Delivery*.

[31] Polyak S. J., Morishima C., Shuhart M. C., Wang C. C., Liu Y., Lee D. Y. W. (2007). Inhibition of T-cell inflammatory cytokines, hepatocyte NF-*κ*B signaling, and HCV infection by standardized silymarin. *Gastroenterology*.

[32] Zhou G. Q., Zhao H. Y., Lu C. (2009). Effect of Ganoderma lucidum polysaccharides on intestinal mucosal immune system in H22 liver cancer bearing mice. *Zhongguo Zhong Xi Yi Jie He Za Zhi*.

[33] He X., Li X., Liu B., Xu L., Zhao A., Lu A. (2011). Down-regulation of Treg cells and up-regulation of TH1/TH2 cytokine ratio were induced by polysaccharide from radix glycyrrhizae in H22 hepatocarcinoma bearing mice. *Molecules*.

[34] Wang M., Wang H., Tang Y. (2011). Effective inhibition of a strongylocentrotus nudus eggs polysaccharide against hepatocellular carcinoma is mediated via immunoregulation in vivo. *Immunology Letters*.

[35] Long S., GuangZhi Y., BaoJie G. (2012). Shikonin derivatives protect immune organs from damage and promote immune responses in vivo in tumour-bearing mice. *Phytotherapy Research*.

[36] Wang J.-M., Ji L.-L., Branford-White C. J. (2012). Antitumor activity of Dioscorea bulbifera L. rhizome in vivo. *Fitoterapia*.

[37] Ge G.-f., Yu C.-h., Yu B., Shen Z.-h., Zhang D.-l., Wu Q.-f. (2012). Antitumor effects and chemical compositions of eupolyphaga sinensis walker ethanol extract. *Journal of Ethnopharmacology*.

[38] Li Q., Bao J. M., Li X. L., Zhang T., Shen X. H. (2012). Inhibiting effect of astragalus polysaccharides on the functions of CD4 + CD25 highTreg cells in the tumor microenvironment of human hepatocellular carcinoma. *Chin Med J (Engl)*.

[39] Shu G., Yang T., Wang C., Su H., Xiang M. (2013). Gastrodin stimulates anticancer immune response and represses transplanted H22 hepatic ascitic tumor cell growth: involvement of NF-*κ*B signaling activation in CD4 +  T cells. *Toxicology and Applied Pharmacology*.

[40] Huang Z., Wang Y., Chen J., Wang R., Chen Q. (2013). Effect of Xiaoaiping injection on advanced hepatocellular carcinoma in patients. *Journal of Traditional Chinese Medicine*.

[41] Yang J., Li X., Xue Y., Wang N., Liu W. (2014). Anti-hepatoma activity and mechanism of corn silk polysaccharides in H22 tumor-bearing mice. *International Journal of Biological Macromolecules*.

[42] Wu R., Ru Q., Chen L., Ma B., Li C. (2014). Stereospecificity of ginsenoside Rg3 in the promotion of cellular immunity in hepatoma H22-bearing mice. *Journal of Food Science*.

[43] Wang Q.-S., Gao L.-N., Zhu X.-N. (2019). Co-delivery of glycyrrhizin and doxorubicin by alginate nanogel particles attenuates the activation of macrophage and enhances the therapeutic efficacy for hepatocellular carcinoma. *Theranostics*.

[44] Han L., Yao S., Cao S. (2019). Triterpenoid saponins from anemone flaccida suppress tumor cell proliferation by regulating MAPK, PD1/PDL1, and STAT3 signaling pathways and altering cancer metabolism. *OncoTargets and Therapy*.

[45] Fan Y., Li S., Ding X. (2019). First-in-class immune-modulating small molecule Icaritin in advanced hepatocellular carcinoma: preliminary results of safety, durable survival and immune biomarkers. *BMC Cancer*.

[46] Lee S. J., Saiki I., Hayakawa Y., Nunome S., Yamada H., Kim S. H. (2003). Antimetastatic and immunomodulating properties of a new herbal prescription, Bojung-bangam-tang. *International Immunopharmacology*.

[47] Li J., Xie M., Gan Y. (2008). Effect of Xiaochaihu decoction and different herbal formulation of component on inhibiting H22 liver cancer in mice and enhancing immune function. *Zhongguo Zhong Yao Za Zhi*.

[48] Cao Z. Y., Chen X. Z., Liao L. M. (2011). Fuzheng yiliu granule inhibits the growth of hepatocellular cancer by regulating immune function and inducing apoptosis in vivo and in vitro. *Chinese Journal of Integrative Medicine*.

[49] Zhao H. J., Du J., Chen X. (2012). Clinical study of Fuzheng Yiliu recipe combined with microwave ablation on hepatocellular carcinoma. *Zhongguo Zhong Xi Yi Jie He Za Zhi*.

[50] Cao Z., Liao L., Chen X. (2013). Enhancement of antitumor activity of low-dose 5-fluorouracil by combination with Fuzheng-Yiliu granules in hepatoma 22 tumor-bearing mice. *Integrative Cancer Therapies*.

[51] Liu X., Li N. (2012). Regularity analysis on clinical treatment in primary liver cancer by traditional Chinese medicine. *Zhongguo Zhong Yao Za Zhi*.

[52] Zhang Q. B., Meng X. T., Jia Q. A. (2016). Herbal compound Songyou yin and moderate swimming suppress growth and metastasis of liver cancer by enhancing immune function. *Integrative Cancer Therapies*.

[53] Zhu Y. C., Zhan G. J., Yuan D. P., Yang F. M., Huang Q. (2017). Effects of shiquanyuzhentang on immunologic function of H22 tumor-bearing mouse. *Zhongguo Ying Yong Sheng Li Xue Za Zhi*.

[54] Chen X., Cao Z., Zhang Y. (2017). Fuzheng qingjie granules inhibit growth of hepatoma cells via inducing mitochondria-mediated apoptosis and enhancing immune function. *Integrative Cancer Therapies*.

[55] Chuang M. H., Chang J. T., Hsu L. J., Jan M. S., Lu F. J. (2017). Antitumor activity of the Chinese medicine JC-001 is mediated by immunomodulation in a murine model of hepatocellular carcinoma. *Integrative Cancer Therapies*.

[56] Fu B., Zhai X., Xi S. (2019). Safety evaluation of a new traditional Chinese medical formula, ciji-hua’ai-baosheng II formula, in adult rodent models. *Evidence-Based Complementary and Alternative Medicine*.

[57] Fu B., Xi S., Wang Y. (2018). The protective effects of ciji-hua’ai-baosheng II formula on chemotherapy-treated H22 hepatocellular carcinoma mouse model by promoting tumor apoptosis. *Frontier Pharmacology*.

[58] Xi S., Fu B., Loy G. (2018). The effects of Ciji-Hua’ ai-Baosheng on immune function of mice with H22 hepatocellular carcinoma receiving chemotherapy. *Biomedical Pharmacotheraphy*.

[59] Lu X., Wo G., Li B. (2018). The anti-inflammatory NHE-06 restores antitumor immunity by targeting NF-kappaB/IL-6/STAT3 signaling in hepatocellular carcinoma. *Biomedical Pharmacotheraphy*.

[60] Zhou J. Y., Chen M., Wu C. E., Zhuang Y. W., Chen Y. G., Liu S. L. (2019). The modified Si-Jun-Zi Decoction attenuates colon cancer liver metastasis by increasing macrophage cells. *BMC Complementary Alternative Medicine*.

[61] Xu H., Wei W., Dong C. (2020). Efficacy and safety of Chinese patent medicine (Jinlong capsule) in the treatment of advanced hepatocellular carcinoma: a meta-analysis. *Bioscience Reports*.

[62] Zhang H. J., Yang J. J., Wang W. X. (2008). Effects of Jinlong Capsule on expressions of interleukin-2 and soluble interleukin-2 receptor in patients with primary liver cancer after transarterial chemoembolization therapy. *Zhong Xi Yi Jie He Xue Bao*.

[63] Yuan Q., Yao F., Zhou L. (2019). Yu ping feng san exert anti-angiogenesis effects through the inhibition of TSLP-STAT3 signaling pathways in hepatocellular carcinoma. *Evid Based Complementary Alternative Medicine*.

[64] Sutti S., Albano E. (2020). Adaptive immunity: an emerging player in the progression of NAFLD. *Nature Reviews Gastroenterology & Hepatology*.

[65] Lu C., Rong D., Zhang B. (2019). Current perspectives on the immunosuppressive tumor microenvironment in hepatocellular carcinoma: challenges and opportunities. *Molecular Cancer*.

[66] Macek Jilkova Z., Kurma K., Decaens T. (2019). Animal models of hepatocellular carcinoma: the role of immune system and tumor microenvironment. *Cancers (Basel)*.

[67] Guo Y. M., Huang Y. X., Shen H. H. (2015). Efficacy of compound kushen injection in relieving cancer-related pain: a systematic review and meta-analysis. *Evidence-Based Complementary and Alternative Medicine*.

[68] Tao W., Luo X., Cui B. (2015). Practice of traditional Chinese medicine for psycho-behavioral intervention improves quality of life in cancer patients: a systematic review and meta-analysis. *Oncotarget*.

[69] Del Corno M., Gessani S., Conti L. (2020). Shaping the innate immune response by dietary glucans: any role in the control of cancer?. *Cancers (Basel)*.

[70] Wang F., Tipoe G. L., Yang C. (2018). Lycium barbarum polysaccharide supplementation improves alcoholic liver injury in female mice by inhibiting stearoyl-CoA desaturase 1. *Molecular Nutrition & Food Research*.

[71] Yang J. H., Lee E., Lee B., Cho W. K., Ma J. Y., Park K. I. (2018). Ethanolic extracts of Artemisia apiacea hance improved atopic dermatitis-like skin lesions in vivo and suppressed TNF-alpha/IFN-Gamma(-)Induced proinflammatory chemokine production in vitro. *Nutrients*.

[72] Cao Z., Liu C., Bai Y. (2017). Inhibitory effect of dihydroartemisinin on chondrogenic and hypertrophic differentiation of mesenchymal stem cells. *American Journal of Translational Research*.

[73] Chen J., Chen J., Wang X., Liu C. (2014). Anti-tumour effects of polysaccharides isolated from artemisia annual by inducing cell apoptosis and immunomodulatory anti-hepatoma effects of polysaccharides. *African Journal of Traditional, Complementary and Alternative Medicines*.

[74] Dong G. H., Wang J., Zhang Y. H. (2012). Induction of p53-mediated apoptosis in splenocytes and thymocytes of C57BL/6 mice exposed to perfluorooctane sulfonate (PFOS). *Toxicology and Applied Pharmacology*.

[75] Chen X., Nie W., Yu G. (2012). Antitumor and immunomodulatory activity of polysaccharides from Sargassum fusiforme. *Food Chemical Toxicology*.

[76] Min R., Tong J., Wenjun Y. (2008). Growth inhibition and induction of apoptosis in human oral squamous cell carcinoma Tca-8113 cell lines by Shikonin was partly through the inactivation of NF-kappaB pathway. *Phytotherapy Research*.

[77] Gao H., Kuroyanagi M., Wu L., Kawahara N., Yasuno T., Nakamura Y. (2002). Antitumor-promoting constituents from Dioscorea bulbifera L. in JB6 mouse epidermal cells. *Biological and Pharmaceutical Bulletin*.

[78] Tang Y., Xue Y. B., Zhou L. (2014). New norclerodane diterpenoids from the tubers of Dioscorea bulbifera. *Chemical and Pharmaceutical Bulletin*.

[79] Liu H., Chou G. X., Guo Y. L., Ji L. L., Wang J. M., Wang Z. T. (2010). Norclerodane diterpenoids from rhizomes of Dioscorea bulbifera. *Phytochemistry*.

[80] Yang F., Liang Y., Xu L. (2016). Exploration in the cascade working mechanisms of liver injury induced by total saponins extracted from Rhizoma Dioscorea bulbifera. *Biomedical Pharmacotherapy*.

[81] Liu H., Chou G. X., Wu T. (2009). Steroidal sapogenins and glycosides from the rhizomes of Dioscorea bulbifera. *Journal of Natural Products*.

[82] Liu H., Chou G. X., Wang J. M., Ji L. L., Wang Z. T. (2011). Steroidal saponins from the rhizomes of Dioscorea bulbifera and their cytotoxic activity. *Planta Med*.

[83] Zhang N., Zhao Y., Shi Y., Chen R., Fu X., Zhao Y. (2019). Polypeptides extracted from Eupolyphaga sinensis walker via enzymic digestion alleviate UV radiation-induced skin photoaging. *Biomed Pharmacother*.

[84] Dai B., Qi J., Liu R., Zhang Y. (2014). Eupolyphaga sinensis Walker demonstrates angiogenic activity and inhibits A549 cell growth by targeting the KDR signaling pathway. *Molecular Medicine Reports*.

[85] Zhan Y., Zhang H., Liu R., Wang W., Qi J., Zhang Y. (2016). Eupolyphaga sinensis walker ethanol extract suppresses cell growth and invasion in human breast cancer cells. *Integrative Cancer Therapies*.

[86] Braumuller H., Wieder T., Brenner E. (2013). T-helper-1-cell cytokines drive cancer into senescence. *Nature*.

[87] Yee D., Shah K. M., Coles M. C., Sharp T. V., Lagos D. (2017). MicroRNA-155 induction via TNF-alpha and IFN-gamma suppresses expression of programmed death ligand-1 (PD-L1) in human primary cells. *Journal of Biological Chemistry*.

[88] Li N., Wang J., Zhang N. (2018). Cross-talk between TNF-alpha and IFN-gamma signaling in induction of B7-H1 expression in hepatocellular carcinoma cells. *Cancer Immunology, Immunotherapy*.

[89] Wang K., Zhang H., Han Q. (2019). Effects of astragalus and ginseng polysaccharides on growth performance, immune function and intestinal barrier in weaned piglets challenged with lipopolysaccharide. *Journal of Animal Physiology and Animal Nutrition*.

[90] Li W., Hu X., Wang S. (2020). Characterization and anti-tumor bioactivity of astragalus polysaccharides by immunomodulation. *International Journal of Biological Macromolecules*.

[91] Li S., Wang X. F., Ren L. N. (2019). Protective effects of gamma-irradiated Astragalus polysaccharides on intestinal development and mucosal immune function of immunosuppressed broilers. *Poultry Science*.

[92] Rezalotfi A., Ahmadian E., Aazami H., Solgi G., Ebrahimi M. (2019). Gastric cancer stem cells effect on Th17/treg balance; A bench to beside perspective. *Frontier Oncology*.

[93] Fu J., Xu D., Liu Z. (2007). Increased regulatory T cells correlate with CD8 T-cell impairment and poor survival in hepatocellular carcinoma patients. *Gastroenterology*.

[94] Fu J., Zhang Z., Zhou L. (2013). Impairment of CD4+ cytotoxic T cells predicts poor survival and high recurrence rates in patients with hepatocellular carcinoma. *Hepatology*.

[95] Hong Y. K., Wu H. T., Ma T., Liu W. J., He X. J. (2009). Effects of Glycyrrhiza glabra polysaccharides on immune and antioxidant activities in high-fat mice. *International Journal of Biological Macromolecules*.

[96] Zhang X., Yang H., Yue S. (2017). The mTOR inhibition in concurrence with ERK1/2 activation is involved in excessive autophagy induced by glycyrrhizin in hepatocellular carcinoma. *Cancer Medicine*.

[97] Bilzer M., Roggel F., Gerbes A. L. (2006). Role of Kupffer cells in host defense and liver disease. *Liver International*.

[98] Alexander D. (2004). A method for interdicting the development of severe jaundice in newborns by inhibiting the production of bilirubin. *Pediatrics*.

[99] Horst A. K., Tiegs G., Diehl L. (2019). Contribution of macrophage efferocytosis to liver homeostasis and disease. *Frontier Immunology*.

[100] Shwartz A., Goessling W., Yin C. (2019). Macrophages in zebrafish models of liver diseases. *Frontier Immunology*.

[101] Zhang Y., He L., Meng L., Luo W. (2008). Taspine isolated from Radix et Rhizoma Leonticis inhibits proliferation and migration of endothelial cells as well as chicken chorioallantoic membrane neovascularisation. *Vascular Pharmacology*.

[102] Han L. T., Li J., Huang F., Yu S. G., Fang N. B. (2009). Triterpenoid saponins from Anemone flaccida induce apoptosis activity in HeLa cells. *Journal of Asian Natural Products Research*.

[103] Oshima H., Oshima M., Inaba K., Taketo M. M. (2004). Hyperplastic gastric tumors induced by activated macrophages in COX-2/mPGES-1 transgenic mice. *EMBO*.

[104] Haftchenary S., Avadisian M., Gunning P. T. (2011). Inhibiting aberrant Stat3 function with molecular therapeutics: a progress report. *Anticancer Drugs*.

[105] Xi S., Hong R., Huang J. (2014). Effects of Ciji Hua’ ai Baosheng granule formula (CHBGF) on life time, pathology, peripheral blood cells of tumor chemotherapy model mouse with H22 hepatoma carcinoma cells. *African Journal of Traditional, Complementary and Alternative Medicines*.

[106] Lee S. T. H. (2020). Inflammation, depression, and anxiety disorder: a population-based study examining the association between Interleukin-6 and the experiencing of depressive and anxiety symptoms. *Psychiatry Research*.

[107] Ohs I., Ducimetiere L., Marinho J., Kulig P., Becher B., Tugues S. (2017). Restoration of natural killer cell antimetastatic activity by IL12 and checkpoint blockade. *Cancer Research*.

[108] Cai J., Wang H., Zhou S., Wu B., Song H. R., Xuan Z. R. (2008). Effect of Sijunzi Decoction and enteral nutrition on T-cell subsets and nutritional status in patients with gastric cancer after operation: a randomized controlled trial. *Zhong Xi Yi Jie He Xue Bao*.

[109] Chen L., Jin T., Ning C., Wang S., Wang L., Lin J. (2019). Anti-tumor and immune-modulating effect of Jiawei Sijunzi decoction in mice bearing hepatoma H22 tumor. *Nan Fang Yi Ke Da Xue Xue Bao*.

[110] Pan B., Cheng T., Nan K. J., Qiu G. Q., Sun X. C. (2005). Effect of Fuzheng Yiliu decoction combined with chemotherapy on patients with intermediate and late stage gastrointestinal cancer. *World Journal of Gastroenterology*.

[111] Wang Y., Shen Y., Wang S., Shen Q., Zhou X. (2018). The role of STAT3 in leading the crosstalk between human cancers and the immune system. *Cancer Letters*.

[112] Wang T., Niu G., Kortylewski M. (2004). Regulation of the innate and adaptive immune responses by Stat-3 signaling in tumor cells. *Nature Med*.

[113] Park E. J., Lee J. H., Yu G. Y. (2010). Dietary and genetic obesity promote liver inflammation and tumorigenesis by enhancing IL-6 and TNF expression. *Cell*.

[114] Liu Y. J., Soumelis V., Watanabe N. (2007). TSLP: an epithelial cell cytokine that regulates T cell differentiation by conditioning dendritic cell maturation. *Annu Rev Immunol*.

[115] Wang Y., Fan D. X., Duan J., Li M. Q., Zhu X. Y., Jin L. P. (2012). Thymic stromal lymphopoietin downregulates NME1 expression and promotes invasion in human trophoblasts via the activation of STAT3 signaling pathway. *Clinical Immunology*.

[116] Ying S., O’ Connor B., Ratoff J. (2005). Thymic stromal lymphopoietin expression is increased in asthmatic airways and correlates with expression of Th2-attracting chemokines and disease severity. *Journal of Immunology*.

[117] Chiu S. C., Lai Y. L., Chang H. H. (2009). The therapeutic effect of modified Yu Ping Feng San on idiopathic sweating in end-stage cancer patients during hospice care. *Phytotherapy Research*.

[118] Tang K. Y., Du S. L., Wang Q. L., Zhang Y. F., Song H. Y. (2020). Traditional Chinese medicine targeting cancer stem cells as an alternative treatment for hepatocellular carcinoma. *Journal of Integrative Medicine*.

